# Diet in Typhoid Fever

**Published:** 1900-01-20

**Authors:** 


### Hospital Clinics and Medical Progress.

### DIET IN TYPHOID FEVER.

Dr. R. W. Marsden,[1] medical superintendent of the Monsall Fever Hospital, puts in a strong plea for the use of less restricted diet in typhoid fever than is generally prescribed at the present day, and to show the advantages of the method which he advocates, he records the results in 200 unselected cases of the disease. By unselected he means all the cases which were admitted during the acute febrile period. The diet made use of has not been markedly different from that commonly employed, but the patients have been more rapidly advanced than is usually done. All the patients of the series received milk only at first, and passed through the usual dietetic stages with greater or less rapidity according to their condition, The patient in a mild case without contra-indication, would receive on successive days bread and milk with custard, fish with mashed potato, chicken, bread and butter, and finally minced meat, remaining at this stage until convalescence was well established; whereas in a severe case peptonised milk alone, or with meat juice, &c., might have to be continued well into the period of convalescence. Speaking broadly, the patient and his condition were alone considered, and slight exacerbations of temperature or occasional irregularities of bowels were not allowed, *per se*, to cause the withdrawal of the food when no evidence existed of a distinct causal connection. Appetite was to a certain extent the guide, and in no case was food forced upon a patient. From a review of his 200 cases, Dr. Marsden concludes that a careful system of dieting, such as the above, has no injurious consequences, and that when one considers the benefits obtained, viz., more rapid recovery, diminished risk of surreptitious feeding with possibly harmful substances, lessened tendency to bolt food when allowed, without proper mastication, and finally, lessened tendency to asthenic complications, such as post-typhoid anæmia, gangrene, &c., one must admit that there is no justification for resisting a craving appetite in the manner at present in vogue. This is but an expression of a feeling which is evidently growing among many who have carefully considered this question. Dr. Barrs,[2] of Leeds, has gone so far as to feed his patients with bread and butter, minced meat, and ordinary mixed diet while suffering from typhoid fever, and having a temperature of 104 deg. Fah. In fact, he recommends the giving of solid food whenever the patient "likes it, wishes for it, and enjoys it." Dr. Shattuck and Dr. Fitz, of Boston, U.S.A., have taken up much the same line. Nevertheless, there is no doubt that the general opinion of the profession remains in favour of feeding typhoid fever exclusively with fluid food through the whole of the pyrexial stage. This general feeling was well expressed by Dr. Phillips in a paper read about a year ago before the Harveian Society, when he said: "I think cases do occur with little or no intestinal ulceration in which solid food might be given early without ill-effect, but in the absence of certain means of distinguishing such cases . . . I think liquid food is the only justifiable mode of nourishment in the active stage of typhoid fever." It is against this monotonous doctrine of "liquids only" that the revolt is taking place. Dr. Morris Manges,[3] of New York, returns to the charge with a long article, in which he urges an increase in the diet of typhoid fever patients, and reviews the observations made by various physicians on the subject. It is pointed out that the long duration of typhoid fever makes it highly important that the patient should be well fed; that, no matter how we feed a patient, there will be intestinal peristalsis, and the waste products must pass over the ulcerated areas; and that we should, therefore, feed the patient according to his digestive powers, and not with reference to the fever, bearing in mind that there is no increased danger of irritation from food which leaves no irritating residue, and which cautious trial shows is digested without disturbance.

The diet suggested by Dr. Manges is that made use of by Dr. Shattuck, including the following articles:—

(1) Milk, hot or cold, with or without salt, diluted with lime water, soda water, Apollinaris, or Vichy water; peptogenic and peptonised milk; cream and water (*i.e.*, less albumin); milk with white of egg, buttermilk, koumyss, matzoon, whey; milk with tea, coffee, or cacao.

(2) Soups—beef, veal, chicken, tomato, potato, oyster, mutton, pea, bean, squash; carefully strained and thickened with rice (powdered), arrowroot, flour, milk or cream, egg, barley.

(3) Mellin's food, malted milk, carnopeptone, bovinine, somatose.

(4) Beef juice.

(5) Gruels—strained corn meal, crackers, flour, barley water, toast water, albumen water with lemon juice.

(6) Ice cream.

(7) Eggs, soft boiled, raw, egg-nog.

(8) Finally, minced lean meat, scraped beef; the soft part of raw oysters; soft crackers with milk or broth; soft puddings without raisins; soft toast without crust; blanc mange, wine jelly, apple sauce, and macaroni.

Of course, this is an American diet, and will require some modification and perhaps translation to suit English ideas; but the point raised by it and by the various papers which we have referred to is, we are sure, one of very considerable importance.

A Russian physician, Dr. Bushuyev, goes further, giving his patients rolls and boiled eggs, cutlets, chicken, and pudding. He and his colleague arranged that for a year all the patients admitted to the hospital should be compared on this basis, Dr. Bushuyev giving a mixed diet and Dr. Sartsievich giving only milk and one or two soft boiled eggs, and the former had the lower mortality of the two. He asserts that the general condition of those on mixed diet was far better than that of those on fluids only.

That the majority of patients do well on milk diet is to be freely admitted, and that patients who, while taking milk diet, interpolate a surreptitious meal of an indigestible kind not unseldom come to grief, is recognised by all. But many typhoid patients digest milk only very imperfectly, and it is to be feared that in some such cases milk diet is practically starvation diet, besides being, to those suffering from the disease in a mild form, desperately monotonous. In the early days of nursing, when those in attendance on the sick were untrained, and could only be relied upon to carry out the simplest and most elementary directions, the broad rules of "fluids only" or of "milk alone" were the best that could be adopted under the circumstances. But with trained nurses as our assistants it ought to be possible to do something better, and to attune the diet to the case with greater accuracy than can be done by ordering fluids, and especially milk alone.

[1] Lancet, Jan. 13. [2] Brit. Med. Jour., Jan. 16, 1897. [3] New York Med. Rec., Jan. 6.

